# Screening for intracranial aneurysms in persons ⩾35 years with hypertension and atherosclerotic disease who smoke(d)

**DOI:** 10.1177/23969873231193296

**Published:** 2023-08-10

**Authors:** Liselore A Mensing, Rick J van Tuijl, Gerard A de Kort, Irene C van der Schaaf, Frank L Visseren, Gabriel JE Rinkel, Birgitta K Velthuis, Ynte M Ruigrok

**Affiliations:** 1 UMC Utrecht Brain Centre, Department of Neurology and Neurosurgery, University Medical Centre Utrecht, Utrecht, The Netherlands; 2 Department of Radiology, University Medical Centre Utrecht, Utrecht, The Netherlands; 3 Department of Vascular Medicine, University Medical Centre Utrecht, Utrecht, The Netherlands

**Keywords:** Intracranial aneurysm, screening, smoking, hypertension

## Abstract

**Introduction:**

Lifetime risk of aneurysmal subarachnoid haemorrhage (aSAH) is high (7%) in persons ⩾35 years with hypertension who smoke(d). Whether screening for intracranial aneurysms (IAs) to prevent aSAH is effective in these patients is unknown.

**Patients and methods:**

Participants were retrieved from a cohort of patients with clinically manifest atherosclerotic vascular disease included between 2012 and 2019 at the University Medical Centre Utrecht (SMART-ORACLE, NCT01932671) in whom CT-angiography (CTA) of intracranial arteries was performed. We selected patients ⩾35 years with hypertension who smoke(d). CTAs were reviewed for the presence of IAs by experienced neuroradiologists. Patients with IAs were offered follow-up imaging to detect aneurysmal growth. We determined aneurysm prevalence and developed a diagnostic model for IA risk at screening using multivariable logistic regression.

**Results:**

IA were found in 25 of 500 patients (5.0% prevalence, 95%CI: 3.3%–7.3%). Median 5 year risk of rupture assessed with the PHASES score was 0.9% (IQR: 0.7%–1.3%). During a median follow-up of 57 months (IQR: 39–83 months) no patients suffered from aSAH. Aneurysmal growth was detected in one patient for whom preventive treatment was advised. IA risk at screening ranged between 1.6% and 13.4% with predictors being age, female sex and current smoking.

**Discussion and conclusion:**

IA prevalence in persons ⩾35 years with hypertension and atherosclerotic vascular disease who smoke(d) was 5%. Given the very small proportion of IA that needed preventive treatment, we currently do not advise screening for Caucasian persons older than 35 years of age who smoke and have hypertension in general. Whether screening may be effective for certain subgroups (e.g. women older than 50 years of age) or other ethnic populations should be the subject of future studies.

## Introduction

Subarachnoid haemorrhage (SAH) caused by rupture of an intracranial aneurysm is a devastating subset of stroke with an incidence of 8 per 100,000 persons-years and a lifetime risk of 0.3%.^1,2^ It occurs at relatively young age, with a mean age of 50–55 years, and more often in women than in men with two-third of patients being women.^1,3^ Outcome after aneurysmal SAH (aSAH) is poor; one in three patients die and one in three remain dependent from help of others.^4^ Prevention of aSAH has high potential to reduce the burden of aSAH. Non-invasive screening for intracranial aneurysms (IAs) with imaging can prevent future aSAH by early detection and preventive treatment of the identified IA.^5^ Such screening is already proven to be cost-effective for the group of first-degree relatives of aSAH patients who have a lifetime risk of aSAH of up to 26%^6^ and with IAs identified in 11% at first screening.^5^ To maximize the potential of aSAH prevention, additional groups with an increased aSAH risk in whom screening is also effective should be identified.

As persons ⩾35 years with hypertension and (a history of) smoking also have a lifetime risk of aSAH of up to 7%, these persons may also qualify as suitable candidates for screening.^2^ Patients with clinically manifest atherosclerotic vascular disease, hypertension and smoking have an increased risk for subsequent ischemic and bleeding events.^7^ Therefore, we aimed to determine the yield of screening for unruptured IA’s in these patients and to assess rupture risk, treatment decisions and follow-up of the IA found. In addition, we aimed to develop a diagnostic model to identify patients with a high risk for having an unruptured IA for whom diagnostic imaging of intracranial arteries would be appropriate.

## Patients and methods

### Study population

All patients aged 35 years or older with hypertension who smoked or had a history of smoking at the time of inclusion were retrieved from the SMART-ORACLE study (Clinicaltrials.gov Identifier 01932671) embedded in the UCC-SMART cohort at the University Medical Centre Utrecht (UMCU). The UCC-SMART cohort is an ongoing cohort study including patients aged 18–79 years referred to the UMCU with clinically manifest atherosclerotic vascular disease (coronary artery disease, cardiovascular disease, transient ischemic attack, non-disabling stroke, peripheral artery disease, abdominal aortic aneurysm), or marked risk factors such as diabetes mellitus type 2 or hypertension.^8^ In the SMART-ORACLE substudy, CT-angiography (CTA) visualizing the aortic arch to the intracranial arteries of the circle of Willis was performed between 2012 and 2019. Patients were excluded in case of known renal failure, previous allergic reaction to contrast or other contra-indication for CT-scanning such as pregnancy. A more detailed description of the study protocol has been published previously.^8^ For the current study we additionally excluded patients (1) with a past medical history of aSAH, unruptured IA (UIA), autosomal dominant polycystic kidney disease (ADPKD) or other disease known to predispose for aneurysm development such as Ehlers-Danlos or fibromuscular dysplasia (FMD), (2) with a positive family history for aSAH (defined as a first-degree relative (parent, sibling or child) with aSAH) at time of inclusion in the SMART-ORACLE cohort, and (3) who were previously screened for IA.

### Standard protocol approvals, registrations, and patient consents

The Medical Ethical Review Committee of the UMCU approved the study protocol, and all patients gave written informed consent. The STROBE guideline for observational studies was followed.

### Baseline characteristics

Baseline characteristics were assessed at time of inclusion in the SMART-ORACLE study. Images were acquired using a 256-slice MDCT-scanner (iCT, Philips Healthcare, the Netherlands) on the same day as the baseline characteristics were assessed.^8^ Smoking was categorized as “never,” “former” or “current.” Excessive alcohol consumption was defined as consumption of ⩾21 units per week. Hypertension was defined as a systolic blood pressure ⩾140 mmHg and/or a diastolic blood pressure ⩾90 mmHg and/or the use of antihypertensive medication. Other diseases in the medical history were defined as diagnosed by a physician and/or the use of the medication for the specific disease. Physical exercise per week was converted to Metabolic Equivalent Task units (METs) per week. Length and weight at time of the CTA were used to calculate Body Mass Index (BMI).

### CTA intracranial arteries

The main focus of the initial evaluation of the CTAs was the presence of atherosclerosis in the coronary and carotid arteries. The intracranial arteries were not assessed in detail, but if obvious abnormalities of the intracranial arteries were detected, these were discussed with the patients as well. For our present study three experienced neuro-radiologists (BKV, ICvdS, GAdK) reevaluated the CTAs after a median period of 56 months (IQR 33–73 months) for the presence of intradural saccular UIAs. Each CTA was evaluated by one neuroradiologist and in case of uncertainty, the decision was reached by consensus among all three neuroradiologists. UIA were classified as “definite” or “possible.” Possible UIAs were either UIAs located near the ophthalmic artery where it was unclear if the location was truly intradural, or UIAs that could not be differentiated with certainty from a posterior communicating artery infundibulum. Aneurysm location and size were recorded. The PHASES score was calculated to estimate 5 year rupture risk of the identified UIA.^9^ All patients in whom definite or possible UIAs were discovered and who were aged <75 years and still alive at time of the diagnosis, were contacted and offered follow-up imaging to detect aneurysmal growth, since growth is a known risk factor for aneurysm rupture.^10^ Follow-up imaging was performed with CTA or 3-Tesla TOF-MRA. Growth was defined as an increase in aneurysm diameter of >1 mm.^11^ After follow-up, the appropriate management of the UIAs (preventive treatment vs follow-up imaging to determine potential aneurysmal growth) was determined by a multidisciplinary team consisting of vascular neurologists, neuro-interventional radiologists and vascular neurosurgeons and discussed with the patient. In case follow-up imaging was decided upon, follow-up data up to August 2022 were included in the present study.

### Data analyses

For baseline characteristics, mean values with standard deviation (SD) or median values with interquartile range (IQR) were calculated depending on the distribution of data. We calculated the prevalence of UIA in our cohort both before and after follow-up imaging, by dividing the total number of patients with a positive screen for UIA by the total number of patients screened.

We performed multivariable logistic regression analysis to study the association between candidate predictors and the presence of an UIA at screening. Candidate predictors were pre-specified based on literature: age at screening, female sex, current smoking, excessive alcohol consumption, hyperlipidemia, diabetes, coronary artery disease, physical exercise, hypertension at physical examination and the interaction between female sex and current smoking.^12–15^ All candidate predictors were considered for inclusion in the model, regardless of their association in the univariate analysis. There were no missing data. Backward selection was performed based on Akaike Information Criterion (AIC).^16^ The resulting model was subsequently corrected for overfitting using bootstrapping. The amount of shrinkage was based on the full model with all candidate predictors to reflect the selection of predictors. We examined the performance of the final diagnostic model by determining its discrimination expressed by the C-statistic and corrected this for optimism. The C-statistic indicates to what extent the model could distinguish persons with a positive and a negative screen. We displayed the discrimination graphically with a receiver operating characteristic (ROC) curve. Subsequently, we generated a risk score by dividing the regression coefficients of the predictors in the final model by the smallest regression coefficient, resulting in points for each predictor from the final model. This risk score was displayed as a score chart accompanied by a table showing the mean estimated risk of finding an IA at screening for each score. The high-risk group was defined as an absolute probability of finding an UIA at first screening ⩾10%, based on the UIA prevalence of 11% in the group of persons with two or more affected first-degree relatives with aSAH and/or IA at first screening.^5^ Statistical analyses were performed using R software (version 3.6.2 R Foundation).^17^

## Results

### Study population

From all 522 patients aged 35 years or older with hypertension who smoke(d) included in the SMART-ORACLE study between 2012 and 2019, 500 patients were included in our study. Patients were excluded because of a past medical history of aSAH (*n* = 18) or UIA (*n* = 1), FMD (*n* = 2), ADPKD (*n* = 1). Mean age at time of CTA was 60.1 years (SD 8.6 years) and 19% were women. In 73% of patients, coronary artery disease was the cardiovascular event that led to inclusion in the SMART-ORACLE study. Other baseline characteristics are shown in Table 1.

**Table 1.** Baseline characteristics.

	Positive screen No. (%)	Negative screen No. (%)
Number of patients	25 (5)	475 (95)
Women	8 (32)	87 (18)
Age at screening, mean (SD)	64 (7)	60 (9)
Ethnicity		
North-American/European/Middle Eastern	24 (96)	464 (98)
African South of Sahara	0 (0)	2 (0)
South Asian	0 (0)	1 (0)
South-East Asian	0 (0)	2 (0)
Japanese/Finnish	0 (0)	0 (0)
Other	1 (4)	6 (1)
Educational level		
Primary school	1 (4)	29 (6)
All types of secondary education	14 (56)	250 (53)
Higher vocational education and university	10 (40)	196 (41)
Current smoking	10 (40)	142 (30)
Excessive alcohol consumption (⩾21 U/week)	1 (4)	50 (11)
Medical history		
Hyperlipidemia	24 (96)	468 (99)
Diabetes mellitus	3 (12)	73 (15)
Coronary artery disease	21 (84)	373 (79)
Cerebrovascular disease	1 (4)	74 (16)
Peripheral artery disease	1 (4)	28 (6)
Abdominal aortic aneurysm	0 (0)	22 (5)
Kidney disease	0 (0)	11 (2)
Medication		
Blood pressure-lowering medication	25 (100)	454 (96)
Antiplatelet therapy	23 (92)	431 (91)
Anticoagulants	2 (8)	45 (9)
Lipid-lowering medication	24 (96)	432 (91)
Physical exercise in total MET per week, median (IQR)	36 (20–49)	49 (30–79)
Physical examination		
Systolic blood pressure in mmHg, mean (SD)	129 (16)	131 (15)
Diastolic blood pressure in mmHg, mean (SD)	77 (10)	79 (9)
Hypertension at physical examination	9 (36)	147 (31)
BMI in kg/m^2^, median (IQR)	26 (25–28)^a^	27 (25–30)

BMI: body mass index; IQR: inter quartile range; MET: metabolic equivalent of task; MRA: magnetic resonance angiography; No.: number; SD: standard deviation; U: units.

a ⩽4% Missings.

### Yield of screening

We identified 25 definite and 10 possible UIAs in 35 of the 500 screened patients, resulting in an UIA prevalence of 7.0% (95%CI: 4.9%–9.6%) when not taking into account the follow-up imaging data. Three of the 25 definite UIAs were already diagnosed at initial evaluation of the CTAs (i.e. directly after the CTA was performed). All patients had a single UIA with median size of 2.5 mm (IQR: 2.0–3.2 mm) and median 5 year risk of rupture according to the PHASES score being 0.9% (IQR: 0.7%–1.3%).^9^ Of the 25 patients with a definite UIA, four had died (with a cause of death other than aSAH) and three were not invited for follow-up because of their age (⩾75 years). No patient suffered from an aSAH during follow up. Of the 10 patients with a possible UIA, three were not invited for follow-up because of their age (⩾75 years). Thus, radiological follow-up was performed in 25 patients. After evaluation of this follow-up imaging, seven possible UIAs were now diagnosed as being definite infundibula. Thus, when also considering the follow-up data, in total 25 definite UIAs in 25 of the 500 screened patients were diagnosed, resulting in a 5.0% UIA prevalence (95%CI: 3.3%–7.3%). Median size of the 25 definite UIAs identified was 2.5 mm (IQR: 2.0–3.5 mm) and median 5-year risk of rupture according to the PHASES score was 0.9% (IQR: 0.7%–1.3%) (Table 2).^9^ For all patients with a definite UIA who were invited for follow-up imaging (18/25), at least one radiological follow-up was available. After a median follow-up of 54 months (IQR: 40–83 months), aneurysmal growth of 1 mm was detected in 1 of these 18 (6%) patients and preventive aneurysm treatment (surgical clipping) was advised for this patient. For 12 of these 18 (67%) patients continuation of radiological follow-up was advised, and for the other 5 patients (28%) follow-up was discontinued because of their age.

**Table 2.** Results of screening persons ⩾35 years with hypertension who smoke(d).

	500 Screened persons No. (%)
Patients with aneurysm identified	25 (5)
Patients with multiple aneurysms	0 (0)
Aneurysm size in mm, median (IQR)	3 (2–4)
Aneurysm location	
Internal carotid artery	2 (8)
Anterior communicating artery	5 (20)
Anterior cerebral artery	1 (4)
Pericallosal artery	1 (4)
Middle cerebral artery	9 (36)
Posterior communicating artery	4 (16)
Posterior circulation	3 (12)
PHASES, median % 5 year rupture risk (IQR)	0.9 (0.7–1.3)
Subjects with follow-up imaging	18 (68)
Follow-up imaging in months, median (IQR)	51 (36–82)
Detection of growth during follow-up	1 (4)
Treatment after follow-up	
Watchful waiting	11 (65)
Surgical clipping	1 (6)
Endovascular treatment	0 (0)
End of follow-up because of age	5 (29)

IQR: inter quartile range; No.: number.

### High-risk groups

**Figure 1.**
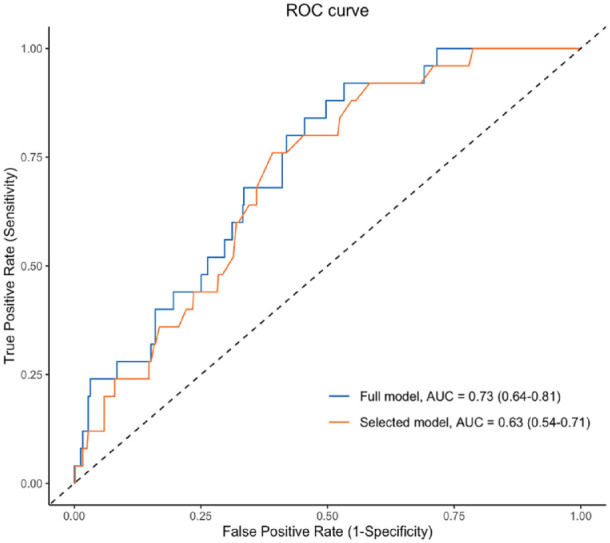
Receiver operating characteristic curve for predicted probability of finding an unruptured intracranial aneurysm at screening.AUC: area under the curve; ROC: receiver operating characteristic.

**Figure 2.**
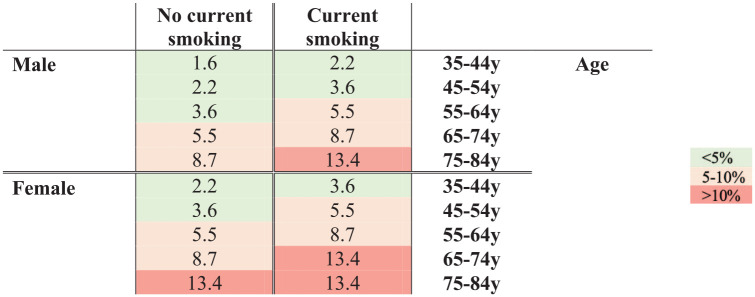
Risk chart with absolute probabilities (%) of finding an unruptured intracranial aneurysm at first screening.y:year.

The full model had a C-statistic of 0.73 (95%CI: 0.64–0.81) and univariate and multivariate ratios for risk of UIA at screening for all candidate predictors are shown in Supplemental Table 1. Multivariable logistic regression identified three predictors for detecting an UIA at screening: age at time of screening, female sex and current smoking (Table 3). After shrinkage, the model had a C-statistic of 0.63 (95%CI: 0.54−0.71) (Figure 1). The regression equation is provided in the legend of Table 3. Regression coefficients were subsequently translated into a score chart (Supplemental Table 2) with the mean predicted probabilities per score shown in Supplemental Table 3. Figure 2 shows a risk chart with estimated probabilities of an UIA at screening in patients aged 35 years or older with hypertension who smoke or had a history of smoking. The mean estimated absolute risk of an IA at screening ranged from 1.6% in men aged 35 to 44 years who never or formerly smoked to the highest estimated risk of 13.4% in men >75 years and women >65 years who currently smoke and in women >75 years who smoked in the past. The patient in whom follow-up imaging detected aneurysmal growth, had an estimated risk of finding an IA at screening of 5.5% (Figure 2).

**Table 3.** Multivariable ratios for risk of unruptured intracranial aneurysms at first screening from the final model before and after shrinkage.

	Multivariate OR (95%CI) before shrinkage	Multivariate OR (95%CI) after shrinkage^a^
Age per year	1.08 (1.03–1.14)	1.05 (1.00–1.11)
Female sex	2.25 (0.89–5.36)	1.70 (0.69–4.22)
Current smoking	2.12 (0.86–5.02)	1.64 (0.68–3.95)

Regression equation: −6.361482 + 0.0510 × Age at time of screening + 0.5333 × Female sex + 0.4934 × Current smoking.

CI: confidence interval; OR: odds ratio.

a Adjusted for optimism with bootstrapping.

## Discussion

In a cohort of patients with clinically manifest atherosclerotic vascular disease, a 5.0% UIA prevalence was found in persons aged 35 years or older with hypertension who smoke or have a history of smoking. No patients suffered from (an aSAH) during follow up. All 25 UIAs identified at screening had a low rupture risk, for which no preventive treatment was advised. Follow-up imaging in 18 patients in the initial years after screening (i.e. patients who were aged <75 years and still alive at time of the UIA diagnosis), showed growth of the UIA in one (6%), and preventive treatment was recommended for this patient. Predictors of a positive first screen for UIA were age at time of screening, female sex and current smoking with predicted IA risk at screening ranging between 1.6% and 13.4% depending on the presence of these predictors.

No other studies on the prevalence of UIAs in persons aged 35 years or more who smoke(d), have hypertension and clinically manifest atherosclerotic vascular disease were identified to directly compare our findings with. However, in the present cohort a higher UIA prevalence was observed than the 3% prevalence in the general population.^15^ A recent prospective pilot study in Finland in which 43 female smokers aged 50–60 years were screened with CTA, found a UIA prevalence of 12% (5/43; 95%CI: 2%–22%) with one of the UIAs found being treated preventively.^18^ However, it cannot be concluded with certainty in this pilot study that the prevalence is higher given the small sample size and the relatively wide corresponding 95%CI.

Previous studies demonstrated female sex to be a risk factor for UIA development in healthy adults,^14,19,20^ with a higher prevalence of UIA in women aged above 50 years.^15^A previous retrospective multicenter case-control study in women aged 30–60 years in whom a MRA was performed, demonstrated that both a history of smoking (OR 3.7, 95%CI: 1.6−8.5) and of hypertension (OR 3.2, 95%CI: 1.2−8.5) were associated with UIA, and that this association became stronger if both risk factors were present (OR 6.9, 95%CI: 2.5−19.2).^21^ In addition, a comparative study performed in Finland showed that hypertension treated with antihypertensive medication was still associated with UIA development.^22^ Our finding that current smokers have an additional risk of UIA development compared with ever smokers, has already been reported in previous prospective population-based studies,^14,20^ and current smoking has also been shown to have an increased risk of aSAH.^23^ Therefore, smoking cessation could be an effective preventive intervention in reducing the risk of growth of UIA and subsequent aSAH. The degree of UIA risk reduction in relation to duration of smoking cessation requires further study. We found evidence that the yield of screening at an older age is probably higher as in our study all persons identified in the high-risk groups were aged over 65 years. On the other hand, screening above the age of 65 has its downsides, because the benefit of preventive treatment of UIA is lower due to a shorter life expectancy and the increasing risk of complications of preventive treatment with age.^24^

A strength of the present study is the uniform data collection at one center, resulting in no missing data on potential predictors of IA development. To reflect clinical practice, the combined prevalence of definite and possible UIAs identified at screening are reported as the diagnosis of both types of UIAs have clinical consequences (i.e. radiological follow-up in possible UIAs). Next, the prevalence of UIAs were reported after integrating the radiological follow-up data which allowed us to exclude some possible UIAs, as these could now be diagnosed with certainty as infundibula.

A few limitations of the study need to be considered. First, our study population included relatively few women. Choosing a population with hypertension and smoking, inevitably leads to a smaller proportion of women, since hypertension and smoking are less prevalent in women than in men. The proportion of women in our study population may be further decreased by the fact that the main reason for inclusion in the SMART-ORACLE cohort was coronary heart disease, which is also more prevalent in men than in women.^23^ Finally, women participate less often in cardiovascular studies such as the SMART-ORACLE cohort than men.^25^ The inclusion of relatively few women most likely resulted in an underestimation of UIA prevalence. In our cohort the prevalence of UIA in women was twice as high as in men, which is comparable to data in the literature.^14,15,18,19^ If hypertension and smoking will be equally prevalent in men and women in the future, aneurysm prevalence in our study domain could increase. Moreover, a lower participation rate of women in our screening cohort will result in an underestimation of aneurysm prevalence. The proportion of participants with a positive screen could increase if we succeed in effectuating a higher participation rate of women in cardiovascular studies. Also, the prevalence of UIA may be different in subgroups of our study population, such as women older than 50 years of age.^15^ Second, hypertension, defined as a systolic blood pressure ⩾140 mmHg and/or a diastolic blood pressure ⩾90 mmHg and/or the use of antihypertensive medication, was one of the inclusion criteria. As beta blockers are prescribed as a risk reduction therapy for patients with coronary vascular disease even in the absence of hypertension,^26^ this may explain the relatively high proportion of patients with coronary artery disease included in this study. This selection bias limits generalizability of our results to all persons aged above 35 years with hypertension who smoke(d), making our prediction model more appropriate to be used in a second-line hospital setting than in first-line general practices. Third, as for the purpose of our study, we included all patients participating in the SMART-ORACLE study, we were not able to select participants based on ethnicity. This resulted in a relatively homogeneous study population regarding ethnicity. Therefore, our findings apply to Caucasian populations and future studies should assess if results are comparable in other populations, including other ethnic populations such as the Finnish and Japanese populations which are known to have an increased risk of aneurysm rupture.^9^ Last, the duration of follow-up was relatively short to draw definite conclusions on risk of growth and rupture of the aneurysms identified. However, the median follow-up time in our study of almost 5 years is the longest described for this population thus far. Final proof should come from long-term follow-up data.

Despite the 5.0% UIA prevalence in this study population, preventive treatment was only advised in 0.2% (i.e. in one patient). In 7.0% of the study population (i.e. 35 patients) follow-up imaging was advised after the first screening. This proportion decreased to 2.4% (i.e. 12 patients) for continued radiological follow-up, as after the first follow-up imaging some possible UIAs could then be diagnosed as being definite infundibula and in five patients follow-up was discontinued because of their age. Given the very small proportion of preventively treated IAs, we currently do not advise screening for UIA in this population. However, this advice may change if more UIA grow during future follow-up imaging, necessitating preventive treatment of these UIAs. Even if we identified more aneurysmal growth in the subgroup with high prevalence, we would still need cost-effectiveness analysis to determine whether screening is effective in these high-prevalence groups. Future studies should assess the effect of smoking cessation on UIA risk.

In conclusion, a 5% IA prevalence was found in persons ⩾35 years with clinically manifest atherosclerotic vascular disease, hypertension and who smoke(d). Given the very small proportion of preventively treated UIA, we currently advise not to screen for this population. Whether screening is effective for certain high-risk groups depends on the risk of growth over time of these IAs and should be the subject of future studies.

## Supplemental Material

sj-docx-1-eso-10.1177_23969873231193296 – Supplemental material for Screening for intracranial aneurysms in persons ⩾35 years with hypertension and atherosclerotic disease who smoke(d) Click here for additional data file. Supplemental material, sj-docx-1-eso-10.1177_23969873231193296 for Screening for intracranial aneurysms in persons ⩾35 years with hypertension and atherosclerotic disease who smoke(d) by Liselore A Mensing, Rick J van Tuijl, Gerard A de Kort, Irene C van der Schaaf, Frank L Visseren, Gabriel JE Rinkel, Birgitta K Velthuis and Ynte M Ruigrok in European Stroke Journal

## References

[1] Etminan N Chang H-S Hackenberg K , et al. Worldwide incidence of aneurysmal subarachnoid hemorrhage according to region, time period, blood pressure, and smoking prevalence in the population: a systematic review and meta-analysis. JAMA Neurol 2019; 76: 588–597. 30659573 10.1001/jamaneurol.2019.0006 PMC6515606

[2] Vlak MH Rinkel GJ Greebe P , et al. Lifetime risks for aneurysmal subarachnoid haemorrhage: multivariable risk stratification. J Neurol Neurosurg Psychiatry 2013; 84: 619–623. 23355806 10.1136/jnnp-2012-303783

[3] Macdonald RL Schweizer TA . Spontaneous subarachnoid haemorrhage. Lancet 2017; 389: 655–666. 27637674 10.1016/S0140-6736(16)30668-7

[4] Vergouwen MD Jong-Tjien-Fa AV Algra A , et al. Time trends in causes of death after aneurysmal subarachnoid hemorrhage: a hospital-based study. Neurology 2016; 86: 59–63. 26590269 10.1212/WNL.0000000000002239

[5] Bor AS Rinkel GJ van Norden J , et al. Long-term, serial screening for intracranial aneurysms in individuals with a family history of aneurysmal subarachnoid haemorrhage: a cohort study. Lancet Neurol 2014; 13: 385–392. 24618352 10.1016/S1474-4422(14)70021-3

[6] Bor AS Rinkel GJ Adami J , et al. Risk of subarachnoid haemorrhage according to number of affected relatives: a population based case-control study. Brain 2008; 131: 2662–2665. 18819992 10.1093/brain/awn187

[7] Kaasenbrood L Boekholdt SM van der Graaf Y , et al. Distribution of estimated 10-year risk of recurrent vascular events and residual risk in a secondary prevention population. Circulation 2016; 134: 1419–1429. 27682883 10.1161/CIRCULATIONAHA.116.021314

[8] Simons PC Algra A van de Laak MF , et al. Second Manifestations of ARTerial disease (SMART) study: rationale and design. Eur J Epidemiol 1999; 15: 773–781. 10608355 10.1023/a:1007621514757

[9] Greving JP Wermer MJ Brown RD Jr , et al. Development of the PHASES score for prediction of risk of rupture of intracranial aneurysms: a pooled analysis of six prospective cohort studies. Lancet Neurol 2014; 13: 59–66. 24290159 10.1016/S1474-4422(13)70263-1

[10] Brinjikji W Zhu YQ Lanzino G , et al. Risk factors for growth of intracranial aneurysms: a systematic review and meta-analysis. AJNR Am J Neuroradiol 2016; 37: 615–620. 26611992 10.3174/ajnr.A4575 PMC7960173

[11] Backes D Rinkel GJE Greving JP , et al. ELAPSS score for prediction of risk of growth of unruptured intracranial aneurysms. Neurology 2017; 88: 1600–1606. 28363976 10.1212/WNL.0000000000003865

[12] Vlak MH Rinkel GJ Greebe P , et al. Independent risk factors for intracranial aneurysms and their joint effect: a case-control study. Stroke 2013; 44: 984–987. 23422088 10.1161/STROKEAHA.111.000329

[13] Feigin VL Rinkel GJ Lawes CM , et al. Risk factors for subarachnoid hemorrhage: an updated systematic review of epidemiological studies. Stroke 2005; 36: 2773–2780. 16282541 10.1161/01.STR.0000190838.02954.e8

[14] Müller TB Vik A Romundstad PR , et al. Risk factors for unruptured intracranial aneurysms and subarachnoid hemorrhage in a prospective population-based study. Stroke 2019; 50: 2952–2955. 31370767 10.1161/STROKEAHA.119.025951

[15] Vlak MH Algra A Brandenburg R , et al. Prevalence of unruptured intracranial aneurysms, with emphasis on sex, age, comorbidity, country, and time period: a systematic review and meta-analysis. Lancet Neurol 2011; 10: 626–636. 21641282 10.1016/S1474-4422(11)70109-0

[16] Royston P Moons KG Altman DG , et al. Prognosis and prognostic research: developing a prognostic model. BMJ 2009; 338: b604. 19336487 10.1136/bmj.b604

[17] R Core Team. R: A language and environment for statistical computing. Vienna: R Foundation for Statistical Computing, 2019.

[18] Huhtakangas J Numminen J Pekkola J , et al. Screening of unruptured intracranial aneurysms in 50 to 60-year-old female smokers: a pilot study. Sci Rep 2021; 11: 23729. 34887429 10.1038/s41598-021-02963-z PMC8660906

[19] Imaizumi Y Mizutani T Shimizu K , et al. Detection rates and sites of unruptured intracranial aneurysms according to sex and age: an analysis of MR angiography-based brain examinations of 4070 healthy Japanese adults. J Neurosurg 2018; 130: 573–578. 29624149 10.3171/2017.9.JNS171191

[20] Can A Castro VM Ozdemir YH , et al. Association of intracranial aneurysm rupture with smoking duration, intensity, and cessation. Neurology 2017; 89: 1408–1415. 28855408 10.1212/WNL.0000000000004419 PMC5649762

[21] Ogilvy CS Gomez-Paz S Kicielinski KP , et al. Cigarette smoking and risk of intracranial aneurysms in middle-aged women. J Neurol Neurosurg Psychiatry 2020; 91: 985–990. 32723730 10.1136/jnnp-2020-323753

[22] Lindgren AE Kurki MI Riihinen A , et al. Hypertension predisposes to the formation of saccular intracranial aneurysms in 467 unruptured and 1053 ruptured patients in Eastern Finland. Ann Med 2014; 46: 169–176. 24579936 10.3109/07853890.2014.883168

[23] Cras TY Bos D Ikram MA , et al. Determinants of the presence and size of intracranial aneurysms in the general population: the Rotterdam Study. Stroke 2020; 51: 2103–2110. 32517578 10.1161/STROKEAHA.120.029296 PMC7306261

[24] Etminan N Rinkel GJ . Unruptured intracranial aneurysms: development, rupture and preventive management. Nat Rev Neurol 2017; 13: 126–713. 28145447 10.1038/nrneurol.2017.14

[25] Vogel B Acevedo M Appelman Y , et al. The Lancet women and cardiovascular disease commission: reducing the global burden by 2030. Lancet 2021; 397: 2385–2438. 34010613 10.1016/S0140-6736(21)00684-X

[26] Smith SC Benjamin EJ Bonow RO , et al. AHA/ACCF secondary prevention and risk reduction therapy for patients with coronary and other atherosclerotic vascular disease: 2011 update: a guideline from the American Heart Association and American College of Cardiology Foundation. Circulation 2011; 124: 2458–2473. 22052934 10.1161/CIR.0b013e318235eb4d

