# Supplementary material for: Screening for intracranial aneurysms in persons ⩾35 years with hypertension and atherosclerotic disease who smoke(d)
Source: Eur Stroke J. 2023 Aug 10;8(4):1071–8. doi: 10.1177/23969873231193296 (PMC10683722; doi:10.1177/23969873231193296)
Supplement: sj-docx-1-eso-10.1177_23969873231193296 – Supplemental material for Screening for intracranial aneurysms in persons ⩾35 years with hypertension and atherosclerotic disease who smoke(d) [file sj-docx-1-eso-10.1177_23969873231193296.docx]

**Supplementary material**

**Screening for intracranial aneurysms in persons ≥ 35 years with hypertension and atherosclerotic disease who smoke(d)**

Liselore A Mensing, MD; Rick J van Tuijl, MSc; Gerard A de Kort, MD PhD; Irene C van der Schaaf, MD PhD; Frank L Visseren, MD PhD; Gabriel JE Rinkel, MD PhD; Birgitta K Velthuis^*^, MD PhD; Ynte M Ruigrok^*^, MD PhD, on behalf of the UCC-SMART study group

**Table of content Supplementary Material**

Supplementary Table 1. Univariate and multivariate ratios for risk of unruptured intracranial

aneurysms at screening from the full model……………………………………………..page 1

Supplementary Table 2. Score chart………………………………………………..…....page 2

Supplementary Table 3. Predicted probability (%) of an unruptured intracranial aneurysm at first screening based on the prediction score……………………………………………page 3

**Supplementary Table 1. Univariate and multivariate ratios for risk of unruptured intracranial aneurysms at screening from the full model**

| **Predictor** | **Univariate OR**  **(95% CI)** | **Multivariate OR**  **(95% CI)** |
| --- | --- | --- |
| Age at screening, per year | 1.06 (1.01-1.12) | 1.08 (1**.**03-1**.14**) |
| Female sex | 2.10 (0.88-5.02) | 2.97 (0.87-9.10) |
| Current smoking | 1.56 (0.69-3.56) | 2.69 (0.90-7.68) |
| Excessive alcohol consumption | 0.35 (0.05-2.67) | 0.39 (0.02-1.87) |
| Hyperlipidemia | 0.36 (0.04-3.03) | 0.40 (0.05-8.51) |
| Diabetes | 0.75 (0.22-2.57) | 0.66 (0.15-2.02) |
| Coronary artery disease | 0.70 (0.23-2.07) | 0.54 (0.14-1.61) |
| Physical exercise | 1.00 (0.98-1.01) | 1.00 (0.98-1.01) |
| Hypertension at physical examination | 1.26 (0.54-2.91) | 1.16 (0.46-2.78) |
| Female sex*Current smoking | 1.89 (0.54-6.65) | 0.50 (0.07-3.23) |

CI = confidence interval; OR = Odds Ratio.

**Supplementary Table 2. Score chart**

|  | **No current smoking** | **Current smoking** |  |  |
| --- | --- | --- | --- | --- |
| **Male** | 0 | 1 | **35-44y** | **Age** |
|  | 1 | 2 | **45-54y** |  |
|  | 2 | 3 | **55-64y** |  |
|  | 3 | 4 | **65-74y** |  |
|  | 4 | 5 | **75-84y** |  |
| **Female** | 1 | 2 | **35-44y** |  |
|  | 2 | 3 | **45-54y** |  |
|  | 3 | 4 | **55-64y** |  |
|  | 4 | 5 | **65-74y** |  |
|  | 5 | 6 | **75-84y** |  |

y = year

**Supplementary Table 3. Predicted probability (%) of an unruptured intracranial aneurysm at first screening based on the prediction score**

| **Risk score** | **No.** | **Mean predicted probability (%)** |
| --- | --- | --- |
| 0 | 3 | 1.6 |
| 1 | 63 | 2.2 |
| 2 | 167 | 3.6 |
| 3 | 191 | 5.5 |
| 4 | 66 | 8.7 |
| ≥5 | 10 | 13.4 |

No. = number of patients
